# Genomic Landscape Comparison of Cardiac versus Extra-Cardiac Angiosarcomas

**DOI:** 10.3390/biomedicines11123290

**Published:** 2023-12-12

**Authors:** Livia Gozzellino, Margherita Nannini, Milena Urbini, Carmine Pizzi, Ornella Leone, Barbara Corti, Chiara Baldovini, Francesco Angeli, Alberto Foà, Davide Pacini, Gianluca Folesani, Alice Costa, Teresa Palumbo, Maria Concetta Nigro, Gianandrea Pasquinelli, Annalisa Astolfi, Maria Abbondanza Pantaleo

**Affiliations:** 1 Department of Medical and Surgical Sciences (DIMEC), Alma Mater Studiorum—University of Bologna, 40138 Bologna, Italy; livia.gozzellino2@unibo.it (L.G.); margherita.nannini@unibo.it (M.N.); francesco.angeli7@unibo.it (F.A.); mariaconcetta.nigro2@unibo.it (M.C.N.); maria.pantaleo@unibo.it (M.A.P.); 2 Division of Oncology, IRCCS Azienda Ospedaliero-Universitaria di Bologna, 40138 Bologna, Italy; 3 Biosciences Laboratory, IRCCS Istituto Romagnolo per lo Studio dei Tumori (IRST) “Dino Amadori”, 47014 Meldola, Italy; milena.urbini@irst.emr.it; 4 Unit of Cardiology, Department of Medical and Surgical Sciences, University of Bologna, 40138 Bologna, Italy; carmine.pizzi@unibo.it (C.P.); alberto.foa2@unibo.it (A.F.); 5 Division of Pathology, Cardiovascular and Cardiac Transplant Pathology Unit, IRCCS Azienda Ospedaliero-Universitaria di Bologna, 40138 Bologna, Italy; ornella.leone@aosp.bo.it (O.L.); barbara.corti@aosp.bo.it (B.C.); chiara.baldovini@aosp.bo.it (C.B.); 6 Cardiac Surgery Unit, IRCCS Azienda Ospedaliero-Universitaria di Bologna, 40138 Bologna, Italy; davide.pacini@unibo.it (D.P.); gianluca.folesani2@unibo.it (G.F.); 7 IRCCS Azienda Ospedaliero-Universitaria di Bologna, 40138 Bologna, Italy; alice.costa@aosp.bo.it; 8 Interdepartmental Center Alma Mater Institute on Healthy Planet, Alma Mater Studiorum—University of Bologna, 40138 Bologna, Italy; teresa.palumbo4@unibo.it; 9 Division of Pathology, IRCCS Azienda Ospedaliero-Universitaria di Bologna, 40138 Bologna, Italy; gianandr.pasquinelli@unibo.it

**Keywords:** cardiac angiosarcomas, extra-cardiac angiosarcomas, bioinformatics, whole-transcriptome sequencing

## Abstract

Angiosarcomas (ASs) are rare malignant vascular entities that can affect several regions in our body, including the heart. Cardiac ASs comprise 25–40% of cardiac sarcomas and can cause death within months of diagnosis. Thus, our aim was to identify potential differences and/or similarities between cardiac and extra-cardiac ASs to enhance targeted therapies and, consequently, patients’ prognosis. Whole-transcriptome analysis of three cardiac and eleven extra-cardiac non-cutaneous samples was performed to investigate differential gene expression and mutational events between the two groups. The gene signature of cardiac and extra-cardiac non-cutaneous ASs was also compared to that of cutaneous angiosarcomas (n = 9). *H/N/K-RAS* and *TP53* alterations were more recurrent in extra-cardiac ASs, while *POTE*-gene family overexpression was peculiar to cardiac ASs. Additionally, in vitro functional analyses showed that *POTEH* upregulation conferred a growth advantage to recipient cells, partly supporting the cardiac AS aggressive phenotype and patients’ scarce survival rate. These features should be considered when investigating alternative treatments.

## 1. Introduction

Angiosarcomas (ASs) are malignant soft-tissue tumors characterized by an incidence of 1–2% among all sarcomas and poor prognosis [1,2,3]. This subtype arises from endothelial cells of blood or lymphatic vessels and can affect different areas, including the head, neck, breast, bone, and viscera [3]. The median age at diagnosis ranges between 52 and 67 years old, and the distribution between men and women is analogous [2,4].

Low-grade angiosarcomas present a solid component along with low-grade cytology and vessels with open lumina, while high-grade angiosarcomas display high mitotic rates and atypical cells [5]. Angiosarcomas can also be classified according to their onset. Primary ASs arise de novo, whereas secondary ASs develop following chronic lymphedema or exposure to radiotherapy or vinyl chloride [2,3,6]. Other potential risk factors include UV radiation, arteriovenous fistula, and a compromised immune system [6].

Visceral angiosarcomas comprise cardiac sarcomas, which represent 25–40% of cardiac sarcomas [7]. Cardiac ASs primarily arise in the right atrium and have a higher incidence in men with a 2/3:1 ratio [7,8,9]. Malignant cells can also invade the pericardium and cardiac chambers causing obstruction and, subsequently, heart failure [8]. Additionally, this subgroup is characterized by a high proliferation rate and late diagnosis, since symptoms are aspecific and onset is rare. Thus, patients are usually deceased within months of diagnosis [9]. Surgical resection alone remains the most effective option, especially when a tumor is strictly localized; however, a combination of surgery, chemotherapy, and radiation is crucial to prolong overall survival.

Considering the rarity and the heterogeneity in their possible site of origin, depicting a well-defined AS mutational profile has been challenging. Therefore, in the present study, we evaluated the molecular differences and similarities between cardiac and extra-cardiac angiosarcomas using next-generation sequencing.

## 2. Materials and Methods

From 2005 to 2017, 11 patients diagnosed with primary cardiac sarcomas were identified in our center. This case series comprised several rare subtypes including tree angiosarcomas (27%) [10,11], two of which were suitable for genomic analysis (L359 and L360) (SRA Accession Number: PRJNA896891).

**Figure 1:**
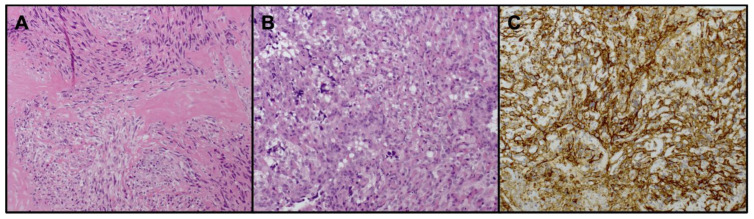
Histological findings representing the L359 and L360 samples. (**A**) L359: the sheet-like proliferation of round to spindle cells associated with focal necrosis and collagen stroma (hematoxylin–eosin, 200×); (**B**) L360: epithelioid pattern with high cell proliferation prevalently made up of epithelioid cells with marked nuclear pleomorphism and intracytoplasmic lumina (hematoxylin–eosin, 200×); and (**C**) L360: immunohistochemistry of neoplastic cells diffusely positive for endothelial marker CD34 (200×).

**Case L359**: A 74-year-old male affected by a right atrial lesion of 7 cm was diagnosed with cardiac MRI and PET-FDG (SUVmax 43.7). He underwent surgery with explorative and diagnostic intent followed by biopsy. The histology was fused, high-grade angiosarcoma (Figure 1). He began one cycle of chemotherapy with doxorubicin and died of the disease after a few weeks. 

**Case L360**: A 39-year-old male affected by a right atrial lesion of 6 cm was diagnosed with a CT scan, MRI, and PET-FDG (SUVmax 8). He underwent tumor resection, which was not completed due to vena cava infiltration. The histology was angiosarcoma (grade 3) with a high mitotic index (14/10 high-power field—HPF) (Figure 1). He began chemotherapy with taxol and died of the disease. 

In addition to these two cases, to evaluate the molecular differences and similarities between cardiac and extra-cardiac angiosarcomas, AS samples from the Gene Expression Omnibus database (https://www.ncbi.nlm.nih.gov/geo/, accessed on 27 September 2022; accession number: GSE163359) were included in this study—one cardiac and twelve extra-cardiac non-cutaneous ASs, whose RNA had been extracted from fresh tissue (Table 1) [12].

### 2.1. Coding Transcriptome Sequencing

Formalin-fixed paraffin-embedded (FFPE) slides were manually macrodissected by a pathologist to achieve a tumor tissue enrichment of at least 70%. Total RNA was extracted using a RecoverAll Total Nucleic Acid Isolation Kit (Thermo Fisher Scientific, Waltham, MA, USA), and cDNA libraries were synthesized from 100 ng total RNA using a TruSeq RNA Exome kit (Illumina, San Diego, CA, USA) following manufacturer’s instructions. cDNA library synthesis included RNA fragmentation, adapter ligation, amplification, and probe hybridization to select coding exon sequences. Subsequently, libraries were sized with Agilent DNA 7500 chips on the Bioanalyzer 2100 (Agilent Technologies, Taipei, Taiwan) and quantified with a fluorometric assay (Quant-IT Picogreen assay; Life Technologies, Carlsbad, CA, USA). Paired-end libraries were sequenced at 80 bp on a NextSeq500 instrument (Illumina, San Diego, CA, USA).

### 2.2. Bioinformatic Analysis

First, quality control of all the FASTQ files was performed with FastQC [13], and the results across all samples were summarized using MultiQC [14]. Two samples (L410 and SRR13260960) were excluded from the analysis due to a high number of overrepresented sequences. Reads of the remaining samples were aligned and mapped on the reference human genome hg38 (No Alts, with decoys) followed by RNA quantification on BaseSpace, an Illumina web tool (http://euc1.sh.basespace.illumina.com/, accessed on 7 October 2022). Small variant calling combined with variant annotation with Nirvana was performed.

Regarding gene alterations, filters were applied to vcf output files to select only stop gain/splice donor/splice acceptor/splice region/frameshift indels/inframe deletion/inframe insertion/initiator codon/ATG loss/missense mutations predicted as deleterious or probably damaging with the SIFT predictor, with a GnomAD frequency <0.01, a QC metric quality >29, a total read depth >14, an alt allele depth >3, and a variant read frequency >0.15. Variants were validated using the Integrative Genomic Viewer (IGV—https://igv.org/, accessed on 10 December 2022). Lastly, only genes belonging to Tier 1 of the Cancer Gene Census list (https://cancer.sanger.ac.uk/census, accessed on 10 December 2022) were considered for further analysis. Sequencing data were analyzed using the DRAGEN RNA app (version 3.10.4). Our mutational events were also compared to the ones from the Angiosarcoma Project—*Count Me In*, with a focus on cutaneous ASs [15]. These additional data (48 samples from 36 patients) were retrieved from the cBioPortal for Cancer Genomics database (https://www.cbioportal.org, accessed on 1 December 2022), including one cardiac angiosarcoma and nine cutaneous ASs of the head, neck, face, and scalp (HNFS). Further analysis was performed using STAR to map paired reads on the reference human genome hg38 [16]; subsequently, duplicate removal, sorting, and indexing were applied using Samtools [17]. Gene expression was quantified and normalized as transcripts per million (TPM) from raw gene counts generated with the Python package HTseq-count [18]. TPM were then used to perform a principal component analysis (PCA) using the R package prcomp [19] and a differential gene expression (DGE) analysis applying the Mann–Whitney *U* test (*p*-value < 0.05), following batch correction with the sva package [20]. Batch correction was necessary since, as previously mentioned, our RNA samples had been extracted from paraffin-embedded tissue, while the GEO samples were derived from fresh tissue. 

A gene set enrichment analysis (GSEA) was performed using the whole expression matrix and selecting the Hallmarks, Reactome, and KEGG gene sets [21]. Moreover, parameters were set as follows: “number of permutations” = “1000”, “permutation type” = “gene set”, “enrichment statistic” = “weighted”, “metric for ranking gene” = “Signal2Noise”, “min size: exclude smaller sets” = “15”, and “normalization mode” = “meandiv”.

### 2.3. Transfection of the POTEH Gene Transcript

To investigate the potential role of *POTEH* overexpression, a plasmid DNA (pcDNA3.1(+)-C-eGFP) containing the whole POTEH transcript under the control of a CMV promoter and upstream of an eGFP-tag sequence was synthesized (GenScript, Rijswijk, NL, USA). The expression plasmid was transformed and amplified in One Shot TOP10 Chemically Competent *E. coli* cells (Life Technologies, Carlsbad, CA, USA) and then purified with an E.Z.N.A. Endo-Free Plasmid DNA Maxi Kit (Omega Bio-tek, Norcross, GA, USA). HEK293 cells were obtained from CLS Cell Lines Service (Eppelheim, Germany) and cultured in Dulbecco’s Modified Eagle Medium high glucose (DMEM, Gibco) enriched with 10% fetal bovine serum (FBS), 1% L-glutamine, and 1% penicillin–streptomycin. Subsequently, the cells were seeded on 6-well plates (0.5 × 10^6^ cells/well) in DMEM 10% FBS without antibiotics, and after 24 h, they were transiently transfected with *POTEH* plasmid or with empty pcDNA3.1 vector using Lipofectamine 2000 (Life Technologies, Carlsbad, CA, USA). Mock-transfected (treated only with Lipofectamine) and pcDNA3.1-transfected cells were used as controls. To assess transfection efficiency, GFP positivity was quantified using a FACSCanto flow cytometer (Beckton Dickinson, Franklin Lakes, NJ, USA). To measure the *POTEH* effect on cell viability, an MTT assay was performed 48 and 72 h after seeding transfected cells in complete medium. Total RNA was extracted 72 h after transfection using the RNeasy Mini Kit (Zymo Research, Irvine, CA, USA), and reverse transcribed with the iScript™ Reverse Transcription Supermix (Bio-Rad, Hercules, CA, USA) into cDNA to quantify *POTEH* overexpression.

### 2.4. Real-Time PCR Analysis

*POTEH* expression was evaluated in transfected HEK293 cells using quantitative RT-PCR on a Roche (LightCycler480, Roche Diagnostics) with a Luna Universal One-Step RT-qPCR kit (New England BioLabs, Ipswich, MA, USA). *GAPDH* and *ATPS* were used as housekeeping genes. GraphPad PRISM Software was used for statistical analysis. The *p*-value was estimated against pcDNA3.1 using an unpaired *t*-test (** *p* < 0.01; *** *p* < 0.001).

## 3. Results

### 3.1. Gene Expression Analysis

**Figure 2:**
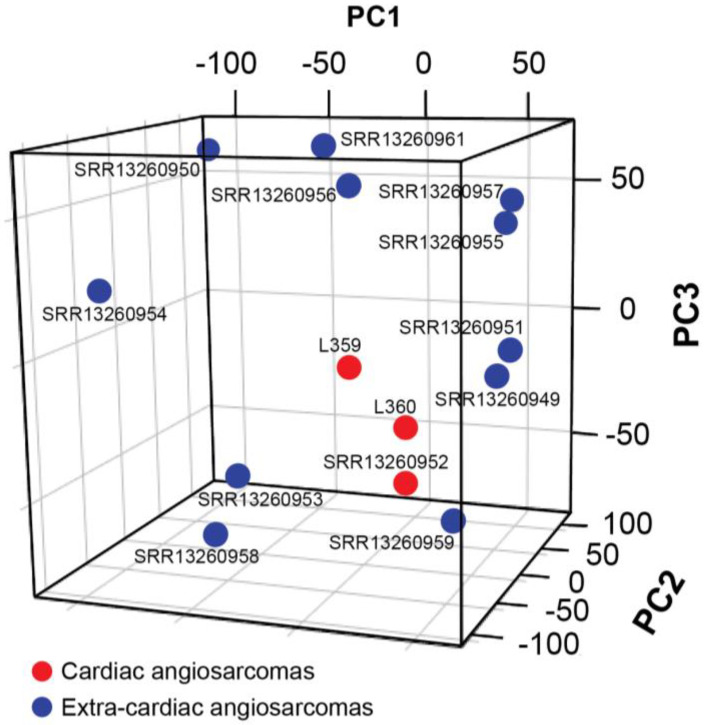
Unsupervised principal component analysis (PCA) on cardiac angiosarcomas (n = 3) versus extra-cardiac non-cutaneous angiosarcomas (n = 11), showing a close localization of cardiac ASs.

First and foremost, the expression profile of three cardiac versus eleven extra-cardiac non-cutaneous ASs was analyzed (Table 1). The unsupervised PCA did not show a distinct cluster of extra-cardiac angiosarcomas along any principal component; however, the three cardiac samples were located close to one another (Figure 2). This suggested the presence of, at least, some common features among cardiac cases and confirmed AS heterogeneity.

**Figure 3:**
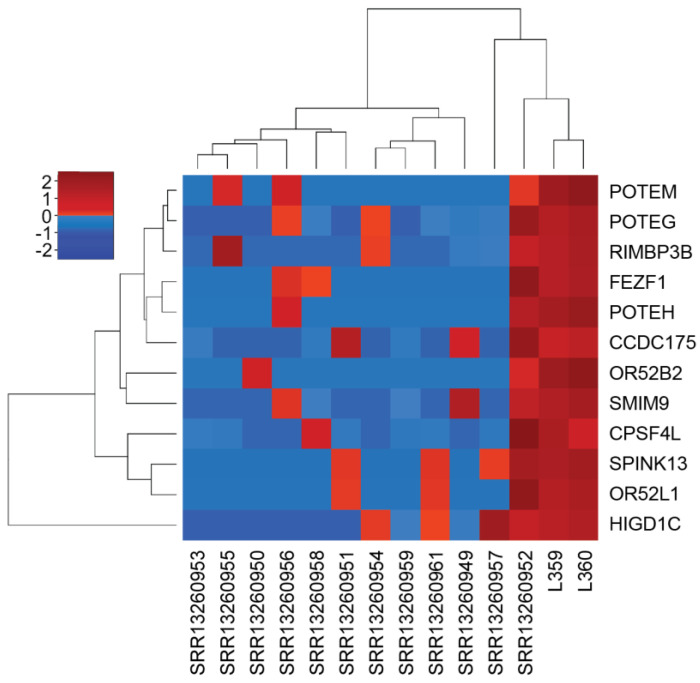
Hierarchical clustering of the most overexpressed gene markers in cardiac (n = 3) versus extra-cardiac non-cutaneous (n = 11) angiosarcomas.

**Figure 4:**
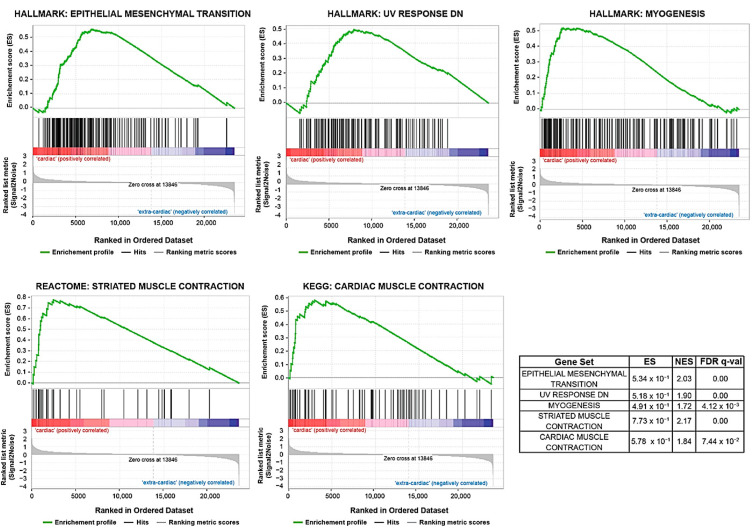
GSEA analysis of enriched pathways in cardiac versus extra-cardiac non-cutaneous angiosarcomas applying the following gene sets: Hallmarks, Reactome, and KEGG. ES: Enrichment Score; NES: Normalized Enrichment Scores; FDR *q*-val: False Discovery Rate *q*-value.

Additionally, a supervised analysis of cardiac versus extra-cardiac angiosarcomas detected 248 differentially expressed genes (*p*-value < 0.05). Interestingly, several *POTE*-family members, including *POTEH*, *POTEG*, and *POTEM*, were upregulated in cardiac ASs (Figure 3). These findings were further validated by the GSEA rank-ordered gene list, where the aforementioned *POTE*-related genes were identified as specific gene markers of cardiac ASs. Moreover, the epithelial–mesenchymal transition (EMT), myogenesis, gene downregulation in response to UV, and cardiac and striated muscle contraction pathways were upregulated in cardiac samples compared with extra-cardiac ASs (GSEA Hallmarks, Reactome, and KEGG databases) (Figure 4).

### 3.2. POTEH Overexpression

**Figure 5:**
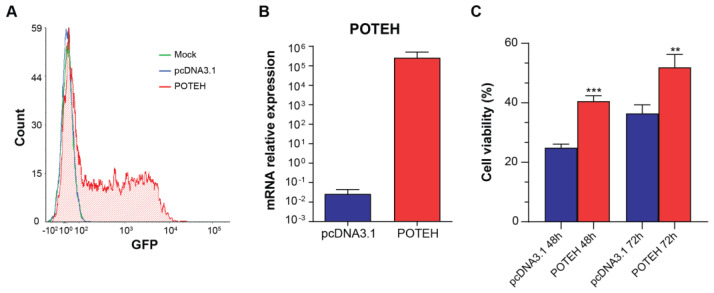
(**A**) GFP expression levels in *POTEH*-transfected cells (red) versus mock- (green) and pcDNA3.1-transfected (blue) cells. (**B**) Higher *POTEH* mRNA expression levels in the *POTEH*-transfected cells compared with pcDNA3.1-transfected cells (RT-PCR). (**C**) MTT assay results showing cell viability in pcDNA3.1- and *POTEH*-transfected cells 48 and 72 h after seeding transfected cells in complete medium (** *p* < 0.01; *** *p* < 0.001).

Due to the significance of *POTE*-family overexpression in cardiac ASs, we decided to investigate the role of *POTE* genes and selected *POTEH* among its family members due to its pronounced upregulation. We transfected HEK293 cells with an expression vector containing the full-length POTEH transcript and an eGFP tag. After 24 h, 49% of *POTEH*-transfected cells exhibited GFP positivity and RT-PCR effectively demonstrated a significant increase in *POTEH* mRNA levels in *POTEH*-transfected cells when compared with pcDNA3.1 controls. Regarding the phenotypic effect, *POTEH* overexpression significantly increased cell viability, with a 63% and 42% increase with respect to pcDNA3.1-transfected cells at 48 and 72 h, respectively (Figure 5). 

### 3.3. Gene Alteration Analysis

First, comparing mutational signatures between cardiac and extra-cardiac non-cutaneous angiosarcomas led to the identification of the following events in ASs of the heart (Table 2). The *RAD51B* missense mutation (Lys243Arg) is classified as pathogenic on COSMIC and probably damaging with a score = 1 on PolyPhen. In contrast, it is considered likely benign and benign on ClinVar and Varsome, respectively. Additionally, *PSIP1* presented a deletion involving four nucleotides (c.651_654del), which caused a frameshift. The *KDR* c.2267-1_2267 insertion shifting the reading frame is defined as likely pathogenic on Varsome; while the *JAK2* point mutation (c.2840G>A) is probably damaging according to PolyPhen and pathogenic with a score = 0.96 on COSMIC.

**Figure 6:**
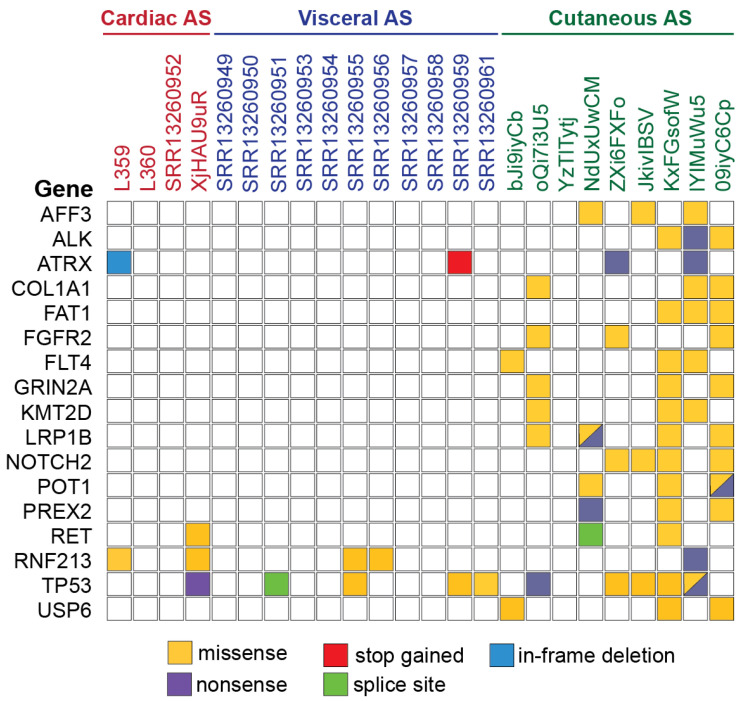
Mutational events classified according to their consequences and occurring in at least 3 samples among cardiac (n = 4), visceral (n = 11), and cutaneous (n = 9) angiosarcomas.

Alterations concerning *RNF213* and *ATRX* were present in both cardiac and extra-cardiac ASs (Figure 6) [15]. Regarding *RNF213*, two point mutations (Ile3318Val and Thr1705Lys) are considered benign on ClinVar and Varsome. According to PolyPhen, the Ile3318Val substitution is also benign, while the other two alterations (Thr1705Lys and Pro2274Leu) are probably damaging with a score of 0.98 and 0.99, respectively. In L359, *ATRX* presented an in-frame deletion (c.6792_6794del) involving exon 31, where a whole triplet encoding glutamic acid was depleted. The same gene presented a point mutation (c.4675A>T) in SRR13260959, causing stop-codon gain. *ATRX* nonsense mutations were also found in two cutaneous samples [15]. 

Including cutaneous AS, the most frequent events concerned *TP53* (n = 11)*. TP53* missense mutations (Arg248Gln, Arg248Trp, Arg280Gly, Tyr220Cys, Pro250Leu, Ser241Phe, and Glu286Lys) are frequently associated with cancer development (Figure 6). For instance, on COSMIC, the Arg248Gln variant is present in 173/3610 mutated samples (4.8%), while Arg248Trp is in 152/3610 (4.2%). The c.920-1G>A splice acceptor variant and the Trp53*, Gln317*, and Gln165* nonsense mutations are classified as pathogenic on ClinVar.

Other remarkable alterations involved the following genes related to tumor onset. *NRAS* and *HRAS* point mutations (Gln61Leu) are considered deleterious on SIFT and possibly damaging with a score = 0.86 and 0.58 on PolyPhen, respectively. On COSMIC, Gln61Leu affecting *NRAS* is observed in 125/1183 (10.6%) mutated samples, while the same variant affecting *HRAS* is observed in 30/234 (12.8%). 

Lastly, the *TSC2* missense mutation (Ile885Val) is of uncertain significance according to ClinVar and Varsome, tolerated on SIFT, and possibly damaging with a score = 0.88 on PolyPhen. The *KMT2C* point mutation (c.5053G>T) is defined as deleterious on SIFT, probably damaging on PolyPhen, and pathogenic with a score = 0.99 on COSMIC, but also as benign on ClinVar. *FANCE* harbored a missense variant (c.1095A>C) classified as deleterious on SIFT and as possibly damaging on PolyPhen. Regarding the *NBN* alteration c.758C>T, there are conflicting interpretations: it is considered deleterious on SIFT, but benign and likely benign on PolyPhen and ClinVar, respectively.

## 4. Discussion

The PCA findings confirmed AS heterogeneity, which partly depends on the tumor site. Indeed, the results also highlighted common features between cardiac and extra-cardiac angiosarcomas.

Among AS-mutated genes, *TP53* is well-known for its role as a tumor suppressor; however, the role of its alterations has remained unclear in cardiac angiosarcomas thus far. A study by Garcia and colleagues and the whole-exome sequencing analysis by Zhrebker’s team did not identify any *TP53* mutations in cardiac ASs [22,23]. In support of this, *TP53* seems to be frequently altered in extra-cardiac ASs, especially in UV-induced head and neck ASs [24,25,26]. In contrast, in a patient affected by cardiac AS, immunohistochemical analyses detected high levels of mutated p53 gene products in tumor cells [22]. Another work showed the presence of *TP53* point mutations mostly in extra-cardiac ASs, but also in two cases of cardiac ASs [27]. These results were further supported by the Angiosarcoma Project [15]: *TP53* was the most altered gene (29.2%), and mutational events occurred in one cardiac AS and in ten extra-cardiac ASs, five of which were cutaneous.

Combining these five cutaneous samples [15] with our findings, *TP53* alterations were identified mainly in extra-cardiac cases (n = 9) and in one cardiac AS. They were missense and truncating variants, which are known for negatively affecting apoptosis, senescence, and DNA repair. Therefore, although our study only included four cardiac ASs, these findings might suggest that *TP53* mutations are, in fact, more common in ASs located outside of the heart.

The second most mutated gene was *RNF213,* which was recently identified as an early-stage lung cancer biomarker [28]. *RNF213* alterations might lead to carcinogenesis because of its involvement in angiogenesis, vasculogenesis, inflammation, and proliferation [29]. In our study and the Angiosarcoma Project [15], this gene was altered in two cardiac ASs and three extra-cardiac ASs. Thus, *RNF213* involvement in AS onset does not seem to strongly correlate with tumor localization.

Alterations affecting the RAS pathway are also remarkable events in AS onset as they can alter cell division, which greatly contributes to tumor development. These signatures are far more common in extra-cardiac ASs [25,26]; indeed, 5/39 extra-cardiac AS cases presented *H/K/N-RAS* mutations [24]. *NRAS* and *HRAS* were exclusively mutated in three and two extra-cardiac cases, respectively [15], while *KRAS* point mutations were only detected in two cases of cardiac ASs [22]. Accordingly, our results showed deleterious *NRAS* and *HRAS* alterations in two extra-cardiac ASs. *NRAS* and *HRAS* are proto-oncogenes involved in cell division; thus, their mutations can lead to uncontrolled proliferation, a feature of cancer cells. Moreover, in the Angiosarcoma Project *NRAS* was mutated in two breast ASs and one cutaneous AS, while *HRAS* was mutated in two ASs of the breast [15].

Unsurprisingly, other genes associated with angiogenesis (*KDR* and *PSIP1*) were altered in our analysis. *KDR*, which encodes a vascular endothelial growth factor (VEGF) receptor, was found mutated in 7–10% of soft-tissue ASs, mainly in the breast [7,15,30]. This was not confirmed in our samples since both breast ASs lacked *KDR* alterations. Conversely, seven out of eight patients presenting *KDR* mutations were diagnosed with breast AS in the Angiosarcoma Project [15]. Furthermore, this gene was mutated in a few cases of cardiac ASs [23,31], as it occurred in one of our cardiac samples (SRR13260952). *KDR* and *PLCG1*, which also contributes to angiogenesis, are mutually exclusive [30]. In this regard, the SRR13260952 case presented solely *KDR* alterations. Mutations in *TP53* and *KDR* are also mutually exclusive [15]; indeed, as previously mentioned, *TP53* alterations only concerned extra-cardiac AS. Both exclusivities were further confirmed in the Angiosarcoma Project [15].

When altered, *PSIP1* can enhance angiogenesis and prevent apoptosis in cancer cells [32]. Additionally, *PSIP1* contributes to homology-directed repair (HDR) [33]. In our study, it is not the only altered gene involved in DNA repair: *RAD51B* participates in homologous recombination repair (HRR) of double-strand DNA breaks [34,35]; *FANCE* is a member of the Fanconi anemia complementation group (FANC) and plays a role in DNA cross-links repair [36]; *NBN* is an MRE11-RAD50-NBN (MRN) component involved in DNA recombination, telomere maintenance, cell division regulation and double-strand DNA break repair [35,36]; and lastly, *KMT2C* is a histone methyltransferase that marks sites for transcription and DNA repair [37]. Genes involved in DNA repair were altered in many extra-cardiac ASs and in one cardiac AS.

Alterations in *ATRX*, *JAK2,* and *TSC2* are also noteworthy since these genes contribute to cell growth and proliferation. *ATRX* is involved in chromatin remodeling, transcription, and telomere maintenance (ALT pathway) [36,38]. In our analysis it was mutated in one cardiac (L359) and in one extra-cardiac case (SRR13260959), while it presented nonsense mutations in two cutaneous ASs. This suggests that *ATRX* alterations might not be subtype-specific biomarkers. *JAK2* plays a major role in growth factor signaling and histone modifications [39,40], while *TSC2* is a tumor suppressor that inhibits cell growth by downregulating the mTORC1 pathway [41]. Consequently, when altered, these genes might cause uncontrolled cell proliferation.

To better distinguish cardiac from extra-cardiac angiosarcomas, we conducted multiple gene expression analyses, whose results will now be explored.

The *POTE*-gene family comprises 14 homologous genes, including cancer-testis antigens (CTAs), located on several chromosomes. These genes are usually upregulated in prostate and epithelial ovarian cancer (EOC), including high-grade serous carcinoma (HGSC) [42,43,44] but also in breast and lung cancer [45]. Their overexpression in ovarian cancer is associated with more advanced stages and poor prognosis [44]. Therefore, *POTE*-related genes might be therapeutically targeted. To validate these observations, we focused on *POTEH*, the most upregulated *POTE* family member in cardiac ASs. This gene is located on chromosome 22 and is expressed in normal prostate, ovarian, and testis tissues and also prostate, ovarian (e.g., HGSC), and lung cancer [43,44,46]. In our research, we transfected mammalian cells with a plasmid containing its transcript sequence and, as a result, successfully transfected cells presented greater growth levels compared with pcDNA3.1-transfected samples. Since *POTEH*-overexpressing cells can be considered more proliferative and resistant over time, gene upregulation in cardiac angiosarcomas highlights a more aggressive phenotype and could at least partly explain patients’ scarce survival rate.

Furthermore, in cardiac sarcomas, we observed an overexpression of genes involved in the epithelial–mesenchymal transition, which is in line with their threatening histotype. In EMT, endothelial cells tend to detach from surrounding cells and, eventually, acquire the phenotype of mesenchymal stem cells, including the ability to migrate and colonize other tissues.

Two common events in angiosarcomas were not detected in our samples. *POT1* is usually mutated in ASs, especially in those located in the head and neck [15,26,47,48]. As proof, it was mutated in three cutaneous cases from the Angiosarcoma Project [15]. Since it is involved in telomere maintenance and apoptosis, *POT1* alterations can lead to longer telomeres and, subsequently, to cell immortality. Mutated *POT1* can enhance *ATR*-dependent DNA damage signaling, which induces cell cycle arrest [48]. Subsequently, cell senescence can cause progenitor cell depletion and tissue stress. Therefore, cells tend to bypass apoptosis and acquire additional somatic mutations, which characterize tumor-aggressive phenotypes.

Another typical event in ASs is *MYC* amplification, especially in secondary angiosarcomas following breast cancer [3,49]. *MYC* is relevant to cancer development because of its involvement in oncogenic signaling. Unfortunately, the whole transcriptome analysis applied in this study did not allow us to verify this event.

Until now, the medical treatment of advanced or unresectable ASs has been independent of tumor localization and comprises the administration of anthracyclines and/or gemcitabine/Taxotere, as stated by consensus conference guidelines [50]. Although our molecular findings did not suggest site-specific variations in the chemotherapy regimen, emerging data regarding cutaneous AS immune sensitivity are intriguing [15,51,52]. Indeed, ipilimumab–nivolumab combined therapy has demonstrated a durable response in ASs, especially in cutaneous tumors of the scalp or face [51]. Additionally, cutaneous angiosarcomas of the head, neck, face, and scalp appeared to be characterized by high tumor mutational burden (TMB) and signatures corresponding to UV light exposure [15,52]. These features suggest that UV damages might greatly contribute to HNFS-AS development. Cutaneous samples from the Angiosarcoma Project presented a greater number of mutational events compared with cardiac and extra-cardiac non-cutaneous ASs, which can be correlated with abundant immune infiltrate [15]. Thus, this AS subtype might be more sensitive to checkpoint inhibitors.

Ultimately, mutational events were more homogeneous in HNFS angiosarcomas than in cardiac ASs, even though both areas are localized. Among the cutaneous samples, the proto-oncogene *RET*, *FGFR2*, and *NOTCH2* involved in cell growth, proliferation, and apoptosis, and *POT1* presented elevated mutational rates, highlighting their role as potential therapeutic targets. Consequently, these differences between cutaneous and non-cutaneous angiosarcomas, and also between cardiac and extra-cardiac ASs, should be carefully considered during treatment selection, although high TMB and UV signature do not ensure promising response to immunotherapy [52].

## 5. Conclusions

Our molecular results supported AS heterogeneity but also highlighted some distinct features between cardiac and extra-cardiac ASs. *TP53* and *H/K/NRAS* mutations seemed to be predominant causative factors in extra-cardiac AS development. Conversely, *POTE* alterations were signatures of cardiac ASs. Since our in vitro results showed that *POTEH* upregulation enhances cell viability, these biomarkers might be relevant to define and, eventually, to predict AS onset in patients.

However, considering the limited sample size as a drawback in our study and more broadly in rare tumor studies, future attempts should be directed toward analyzing more samples to further confirm our findings and potentially identify new alterations. Whole-exome-sequencing analyses should also be performed to investigate gene amplifications and deletions, which might indicate other biomarkers.

From a therapeutic perspective, our results highlighted a higher mutational burden in cutaneous angiosarcomas compared with non-cutaneous ASs. Moreover, since *POTE* genes are cancer-testis antigens, their overexpression in cardiac ASs might suggest their amenability to immunotherapy. This might pave the way to explore alternative targets and treatments in non-cutaneous ASs, especially in angiosarcomas of the heart.

## Figures and Tables

**Table 1:** Sample features.

Sample	Sex	Age	Histotype	Localization
**L359**	male	74	angiosarcoma	heart
**L360**	male	39	angiosarcoma	heart
**SRR13260949**	female	69	angiosarcoma	omentum
**SRR13260950**	male	45	angiosarcoma	lung
**SRR13260951**	female	39	angiosarcoma	perihepatic tissue
**SRR13260952**	male	62	angiosarcoma	heart
**SRR13260953**	female	64	angiosarcoma	breast
**SRR13260954**	unknown	unknown	angiosarcoma	adrenal gland
**SRR13260955**	female	40	angiosarcoma	ovary
**SRR13260956**	female	73	angiosarcoma	left chest wall
**SRR13260957**	male	47	angiosarcoma	lung
**SRR13260958**	female	65	angiosarcoma	breast
**SRR13260959**	male	85	angiosarcoma	liver
**SRR13260961**	male	73	angiosarcoma	soft tissue

**Table 2:** Mutational profile of cardiac (n = 3) and extra-cardiac non-cutaneous (n = 11) angiosarcomas.

Sample	Gene	Chr	cDNA	Protein	Type	COSMIC	ClinVar	Varsome	Gnom- AD Freq.^1^	Alt Allele Depth	Total Read Depth
**L359**	*RAD51B*	14	c.728A>G	p.(Lys243Arg)	missense	pathogenic	lik. Benign ^2^	benign	0.00679	11	19
**L359**	*RNF213*	17	c.9952A>G	p.(Ile3318Val)	missense	NA ^3^	benign	benign	0.00198	14	21
**L359**	*PSIP1*	9	c.651_654del	p.(Ser217Argfs Ter47)	frameshift	NA ^3^	NA ^3^	NA ^3^	NA ^3^	43	64
**L359**	*ATRX*	23	c.6792_6794del	p.(Glu2265del)	inframe	NA ^3^	NA ^3^	NA ^3^	NA ^3^	142	168
**SRR13260952**	*KDR*	4	c.2267-1_2267insTTTACATGTT	p.(Gly756Valfs Ter38)	frameshift	NA ^3^	NA ^3^	lik. Pathog. ^4^	NA ^3^	178	213
**SRR13260952**	*JAK2*	9	c.2840G>A	p.(Arg947Gln)	missense	pathogenic	NA ^3^	uncertain	0.000019	40	76
**SRR13260950**	*KMT2C*	7	c.10763C>T	p.(Ser3588Leu)	missense	pathogenic	benign	uncertain	0.00281	15	29
**SRR13260950**	*TSC2*	16	c.2653A>G	p.(Ile885Val)	missense	NA ^3^	uncertain	uncertain	NA ^3^	56	96
**SRR13260951**	*TP53*	17	c.920-1G>A	NA ^3^	splice acceptor	NA ^3^	pathogenic	uncertain	NA ^3^	32	34
**SRR13260953**	*HRAS*	11	c.182A>T	p.(Gln61Leu)	missense	pathogenic	uncertain	uncertain	NA ^3^	135	332
**SRR13260955**	*TP53*	17	c.743G>A	p.(Arg248Gln)	missense	pathogenic	pathogenic	pathogenic	0.000019	68	120
**SRR13260955**	*RNF213*	17	c.5114C>A	p.(Thr1705Lys)	missense	NA ^3^	benign	benign	0.0045	101	131
**SRR13260955**	*NBN*	8	c.758C>T	p.(Thr253Ile)	missense	NA ^3^	benign	benign	0.000092	71	130
**SRR13260956**	*RNF213*	17	c.6821C>T	p.(Pro2274Leu)	missense	NA ^3^	NA ^3^	benign	0.000019	34	63
**SRR13260956**	*NRAS*	1	c.182A>T	p.(Gln61Leu)	missense	pathogenic	pathogenic	pathogenic	NA ^3^	23	126
**SRR13260957**	*FANCE*	6	c.1095A>C	p.(Arg365Ser)	missense	NA ^3^	uncertain	benign	0.000526	20	36
**SRR13260959**	*TP53*	17	c.659A>G	p.(Tyr220Cys)	missense	pathogenic	pathogenic	pathogenic	0.000007	54	143
**SRR13260959**	*ATRX*	23	c.4675A>T	p.(Lys1559Ter)	stop gain	NA ^3^	NA ^3^	lik. Pathog. ^4^	NA ^3^	22	41
**SRR13260961**	*TP53*	17	c.749C>T	p.(Pro250Leu)	missense	pathogenic	uncertain	pathogenic	NA ^3^	26	68

^1^ Freq. = frequency; ^2^ Lik. Benign = likely benign; ^3^ NA = not applicable; ^4^ Lik. Pathog. = likely pathogenic.

## Data Availability

Publicly available datasets were analyzed in this study. Our data can be found at https://www.ncbi.nlm.nih.gov/sra/PRJNA896891, accessed on 27 September 2022. Data from the Gene Expression Omnibus database can be retrieved from https://www.ncbi.nlm.nih.gov/geo/, accessed on 27 September 2022—accession number: GSE163359, and data from the Angiosarcoma Project—*Count me in* can be found in the cBioPortal for Cancer Genomics database (https://www.cbioportal.org, accessed on 1 December 2022).
